# Synergistic antitumor activity between HER2 antibody-drug conjugate and chemotherapy for treating advanced colorectal cancer

**DOI:** 10.1038/s41419-024-06572-2

**Published:** 2024-03-05

**Authors:** Hongfu Liu, Dongdong Zhou, Dongqin Liu, Xi Xu, Kai Zhang, Ruxia Hu, Peng Xiong, Changxin Wang, Xiangfu Zeng, Liefeng Wang, Shuyong Zhang

**Affiliations:** 1 Department of General Surgery, First Affiliated Hospital, Gannan Medical University, Ganzhou, 341000 China; 2 Key Laboratory of Prevention and Treatment of Cardiovascular and Cerebrovascular Diseases (Ministry of Education), Gannan Medical University, Ganzhou, 341000 China; 3 School of Basic Medicine, Gannan Medical University, Ganzhou, 341000 China

**Keywords:** Targeted therapies, Preclinical research

## Abstract

Colorectal cancer (CRC) is the third most common cancer associated with a poor prognosis. Effective targeted therapy alone or in combination for treating advanced CRC remains to be a major clinical challenge. Here, we propose the therapeutic efficacy and molecular mechanism underlying RC48, a FDA-approved anti-HER2 antibody conjugate via a cleavable linker to the microtubule inhibitor monomethyl auristatin E (MMAE), either alone or in combination with gemcitabine (GEM) in various models of HER2-positive advanced CRC. Our findings demonstrated that HER2 was widely expressed and located on the plasma membrane of CRC patient specimens, PDX xenograft tumors and cell lines. It confirmed that RC48 alone significantly targeted and eradicated HER2 positive CRC tumor in these models. Moreover, we screened a panel of FDA-approved first-line chemotherapy drugs in vitro. We found that GEM exhibited stronger antiproliferative activity compared to the other first-line anti-cancer agents. Furthermore, combination therapy of RC48 and GEM significantly showed synergetic antitumor activity in vitro and in vivo. To gain further mechanistic insights into the combination therapy, we performed RNA-seq analysis. The results revealed that combination treatment of RC48 and GEM regulated multiple signaling pathways, such as PI3K-AKT, MAPK, p53, Foxo, apoptosis, cell cycle and cell senescence, etc., to exert its antitumor activity in CRC cells. Collectively, these preclinical findings demonstrated that RC48 alone or combinational therapy exerted promising antitumor activity, and meriting the preclinical framework for combinational therapy of anti-HER2 drug conjugate drug and chemotherapy drugs for HER2-positive patients with advanced CRC.

## Introduction

Colorectal cancer (CRC) is the third most common cancer and the second most lethal cancer [1, 2]. The 5-year overall survival rate of CRC is about 60%, while that of metastatic CRC is reduced to about 14%. Surgery is no longer effective for advanced CRC, and its refractory and relapse are still the main problems [3]. Currently, chemotherapy remains the main treatment for advanced CRC. However, second- and even third-line treatments often have poor outcomes and overall survival (OS) when first-line chemotherapy regimens failed and tumors relapsed or progressed. With the rapid progress of targeted therapy, many clinical studies have confirmed that chemotherapy combined with targeted drugs can prolong the OS of CRC patients and overcome chemotherapy resistance [2–5]. Therefore, there is still an urgent clinical unmet need to develop effective methods to treat advanced CRC.

Human epidermal growth factor receptor-2 (HER2), is one of the most successfully therapeutic target expressed in various tissues [6–8]. When HER2 gene is amplified and/ or is overexpressed, it may lead to subsequent dysregulated proliferation, differentiation, carcinogenesis and metastasis via interlinked signal transduction including activation of the PI3K/Akt and MAPK pathways, etc [6, 8, 9]. In clinical practice, anti-cancer targeting HER2 can be used to specifically kill tumor cells and minimize the damage to normal cells. Tumor types with amplification or abnormal expression of HER2 include breast cancer, gastric cancer and urothelial cancer, etc., which may potentially benefit from HER2 targeted therapy [6, 8].

Antibody drug conjugate (ADC) is one of the hot research directions in the field of tumor precision therapy in recent years [10–12]. To date, three ADCs targeting HER2, namely T-DM1, T-DXd and RC48, has been successively approved [13, 14]. T-DM1, a first-in-class ADC composed of trastuzumab conjugated via a non-cleavable linker to the tubulin inhibitor DM1, has been approved for the treatment of HER2-positive metastatic breast cancer patients who had previously received trastuzumab and taxane singly or in combination [14–16]. T-DXd is composed of trastuzumab, cleavable linker and cytotoxic topoisomerase I inhibitor deruxtecan [14]. To date, T-DXd is most successful in HER2 positive breast cancer, and it has been pushed forward by the back line treatment of advanced or metastatic HER2-positive breast carcinoma patients [16–19]. RC48, an ADC composed of a humanized anti-HER2 monoclonal antibody (hertuzumab) conjugated via a cleavable linker to the microtubule inhibitor monomethyl auristatin E (MMAE), which have been approved for the treatment of advanced or metastatic HER2-positive gastric cancer and urothelial cancer [20–22]. Preclinical study demonstrated that RC48 exerted much more potent antitumor effects than T-DM1 in HER2-positive breast carcinoma and gastric cancer [20–22]. Thus, it is very important to explore ADC targeting HER2 alone and/or combination treatment of other HER2-positive solid tumors.

Gemcitabine (GEM), 2’, 2’-difluoro-2’-deoxycytidine, is a novel FDA-approved deoxycytidine analog that can kill cells with active DNA synthesis (S phase) and block cell cycle progression at the G1/S phase [23, 24]. GEM is now widely used as an anti-cancer chemotherapeutic drug. And GEM is the most commonly administrated as a single agent for the first-line chemotherapeutic drug for advanced pancreatic cancer [25]. It is also recommended for the treatment of non-small cell lung cancer, breast cancer, bladder cancer and ovarian cancer [26–30]. Many patients have initially benefited from GEM-based chemotherapy but would eventually develop resistance, and the therapeutic efficacy of GEM is limited by the innate and acquired resistance, leading to treatment failure and/or recurrence. Therefore, further exploration of GEM in combination with targeted drugs for treating CRC is necessary.

Herein, we investigated the tumoricidal effects and the molecular mechanism underlying RC48, either alone or in combination with GEM for treating advanced CRC in vitro and in vivo.

## Methods and materials

### Reagents

RC48 was purchased from RemeGenCo., Ltd. GEM, 5-fluorouracil (5-FU), oxaliplatin, etoposide, doxorubicin, cisplatin or carboplatin was purchased from Selleck (Houston, USA). UltraSensitiveTM SP (mouse/rabbit) IHC Kit was purchased from MXB Biotechnology (Fujian, China). Antibody information was shown in Supplementary Table 1.

### Cell lines

COLO205, P53R, RKO and HCT116 were purchased from iCell Bioscience Inc. (Shanghai, China) or the Cell Bank of Chinese Academy of Medical Sciences (Beijing, China). P53R and RKO cells were cultured in MEM medium (Gibco) supplemented with 10% fetal bovine serum (Gibco) and 1% penicillin/streptomycin (Biochem, Shenzhen, China). All the cell identity was validated by short tandem repeats (STR) and confirmed to be free of mycoplasma comtamination. HCT116 cells were cultured in McCoy’s 5A medium (Gibco) supplemented with 10% fetal bovine serum (Gibco) and 1% penicillin/streptomycin (Biochem, Shenzhen, China). COLO205 cell were cultured in RPMI-1640 medium (Gibco) supplemented with 10% fetal bovine serum (Gibco) and 1% penicillin/streptomycin (Biochem, Shenzhen, China) in a humidified incubator (Thermo Fisher Scientific, Waltham, MA) with 5% CO2 at 37 °C.

### In vitro cytotoxicity

CRC cell lines were treated with RC48 and trastuzumab or GEM for three days. Cell viability was determined by Cell Titer-Glo® luminescent cell viability assay kit (Promega, G7572) according to the manufacturer’s instruction. The absorbance was calculated by SPARK Multiplate Reader (TECAN, Switzerland).

### EdU staining assay

COLO205, P53R, RKO and HCT116 cells (1 × 10^5^Cells/well) were seeded on the coverslips in 12-well plates and treated with RC48 for 48 h. After treatments, EDU staining was performed using BeyoClick™ EdU Cell Proliferation Kit (C0075S, Beyotime. Shanghai, China) in accordance with the accompanied instructions manual.

### Cell apoptosis and cycle arrest assay

The CRC cells were incubated in 6-well plates with RC48 and/or GEM for 48 h. For cell apoptosis analysis, cell death was detected by using an Annexin V-FITC apoptosis kit (Beyotime Biotechnology, Shanghai, China). For cell cycle analysis, the cells were stained with propidium iodide (Beyotime Biotechnology, Shanghai, China), and total DNA content was detected with propidium iodide. Then cell cycle and apoptosis were measured by a Flow cytometer (BD, FACSCantoII, USA).

### Clonogenic assay

CRC Cells were seeded in 6-well plates. Next day, cells were treated with RC48 and/or GEM at the different concentrations for 2 weeks. Then cell colonies were washed with PBS, fixed with 4% paraformaldehyde for 30 min at room temperature, and stained with 0.01% (w/v) crystal violet for 30 min at room temperature. The stained colonies were scanned and total colony area was quantitated using Image J software.

### RNA-Seq analysis

Total RNA samples were isolated from P53R and RKO cells, divided into control group (*N* = 3), RC48 (*N* = 3), GEM (*N* = 3) and combinational treated group (Comb; *N* = 3). Total amounts and integrity of RNA were assessed using the RNA Nano 6000 Assay Kit of the Bioanalyzer 2100 system (Agilent Technologies, CA, USA). mRNA was then purified from total RNA by poly-T oligo-attached magnetic beads.The library fragments were purified with AMPure XP system (Beckman Coulter, Beverly, USA). The PCR product was purified by AMPure XP beads, after which the sequencing library was constructed and qualified. The different libraries are pooling according to the effective concentration and the target amount of data off the machine, then being sequenced by the Illumina NovaSeq 6000, and the end reading of 150 bp pairing is generated. The RNA-seq data is available at GEO (GSE255349).

Raw data (raw reads) of fastq format were firstly processed through fastp software. In this step, clean data (clean reads) were obtained by removing reads containing adapter, reads containing ploy-N and low quality reads from raw data. All the downstream analyses were based on the clean data with high quality. FeatureCounts v1.5.0-p3 was used to count the reads numbers mapped to each gene. And then FPKM of each gene was calculated based on the length of the gene and reads count mapped to this gene. Differential expression analysis of two conditions/groups (two biological replicates per condition) was performed using the DESeq2 R package (1.20.0).

Kyoto Encyclopedia of Genes and Genomes (KEGG) enrichment analysis were used clusterProfiler R package to test the statistical enrichment of differential expression genes in KEGG pathways. Gene Set Enrichment Analysis (GSEA) use the local version of the GSEA analysis tool (http://www.broadinstitute.org/gsea/index.jsp). KEGG data set were used for GSEA independently.

### Western blot analysis

CRC cells were incubated with RC48 and/or GEM for two days. Then whole cell lysates were lysed by SDS lysis buffer (Beyotime, Shanghai, China) supplemented with PMSF (Solarbio, Beijing, China) and Phosphatase Inhibitor Cocktail (Beyotime, Shanghai, China). All samples were analyzed by SDS-PAGE and then transferred to PVDF membranes (Millipore, Darmstadt, Germany). The membranes were blocked with 5% bovine serum albumin (Solarbio, Beijing, China) for 1 h at room temperature, and then incubated with the primary antibodies at 4 °C overnight. Then the membranes were incubated with secondary antibodies for 1 h at room temperature. The protein bands were detected using the ECL reagent (Cytiva, USA) and Bio-Rad multifunctional chemiluminescence imaging system.

### In vivo antitumor efficacy in CDX models

BALB/c nude mice (5–6 weeks old) were purchased from SJA Laboratory Animal Co., Ltd. (Hunan, China). All animal experiments were approved and performed in full compliance with guidelines approved by the Biomedical Research Ethics Committee, Gannan Medical University (Jiangxi, China). 5 × 10^6^ COLO205 or HCT116 cells suspended in 100 μL PBS were injected subcutaneously into the right flank of mice. When the tumor volume reached 100 to 150 mm^3^, the mice were randomized into 2 to 4 groups and injected intravenously with RC48 and trastuzumab at various doses with QW×3 manner. Then mice were intravenously received saline, 10 mg/kg RC48, 10 mg/kg trastuzumab. In addition, for the combination therapy study, BALB/c nude mice were treated with vehicle, 2.5 or 5 mg/kg RC48 for QW×3, 10 or 40 mg/kg GEM for BIW×3 and combination with RC48 and GEM. Tumor sizes and body weight were recorded twice a week, and tumor volumes were determined according to the formula: tumor volume (mm^3^) = length × (width)^2^ × 0.5.

### In vivo antitumor efficacy in PDX models

NOD/SCID mice, 5–6-week-old female, which were and provided by Cyagen Biosciences Inc (Suzhou, China). All animal tests were performed in accordance with the guidelines approved by the Biomedical Research Ethics Committee, Gannan Medical University (Jiangxi, China). CRC xenograft tumor tissues were cut into pieces of 2 to 3 mm^3^ and inoculated in the right flank of mice. While the average tumor volumes were reached approximately 100–150 mm^3^ after inoculation, the tumor-bearing mice were randomly divided into 2–4 groups. Tumor-bearing mice were intravenously administrated with vehicle, 10 mg/kg of RC48 once weekly. In addition, for the combination therapy study, NOD/SCID mice were treated with vehicle, 2.5 mg/kg or 5 mg/kg RC48 for QW×3, 10 mg/kg or 40 mg/kg GEM for BIW×3, and combination with RC48 and GEM. Tumor sizes and body weight were recorded twice a week, and tumor volumes were determined according to the formula: tumor volume (mm^3^) = length × (width)^2^ × 0.5.

### TUNEL assay

TdT-UTP nick end-labeling (TUNEL) assay was performed in frozen sections principally according to One Step TUNEL Apoptosis Assay Kit protocol (C1088, Beyotime, Shanghai, China). Briefly, Tumor tissue sections were fixed with 4% paraformaldehyde, then permeabilized by 0.1% Triton X-100 for 10 min. Slides were incubated with TUNEL detection reagent for 60 min at 37 °C. Tissue samples were observed under the laser scanning confocal microscope (Zeiss880, Germany).

### Immunofluorescence

CRC tumor tissue were fixed with 4% paraformaldehyde for 24 h, dehydrated with gradient sucrose, and embedded in O.C.T. Compound and sectioned at 10μm thickness. The slides were washed in PBS and blocked in 5% BSA for 1 hours, incubated HER2 primary antibody with overnight at 4 °C, incubated with Goat anti-Rabbit IgG (H + L) Cross-Adsorbed Secondary Antibody, Alexa Fluor™ 488 for 1 h at room temperature. Images were captured by the laser scanning confocal microscope (Zeiss880, Germany).

### H&E staining and immunohistochemistry

CDXs and PDXs xenograft tumor tissues were fixed with formalin. Paraffin-embedded tissue section of 4 μm for H&E and IHC were stained by a standard histological protocol. And imaging was captured with Leica DM2500 fluorescence Microscope (Leica, Germany).

### Statistical analysis

Results of all experiment were presented as mean values ± standard error of the mean (SEM) by GraphPad Prism 9 software. IC50 values were determined by nonlinear regression analysis of concentration response curves using SPSS 16.0. The statistical significance between two groups were analyzed by one-way ANOVA or Student’s *t* test compared with the untreated or vehicle control group. For all experiments, differences were considered significant for *P* < 0.05 and reported in the figures as follows: *, 0.01 ≤ *P* < 0.05; **, 0.001 ≤ *P* < 0.01; ***, *P* < 0.001.

## Results

### RC48 remarkably inhibited the proliferation of CRC cell lines in vitro

**Fig. 1:**
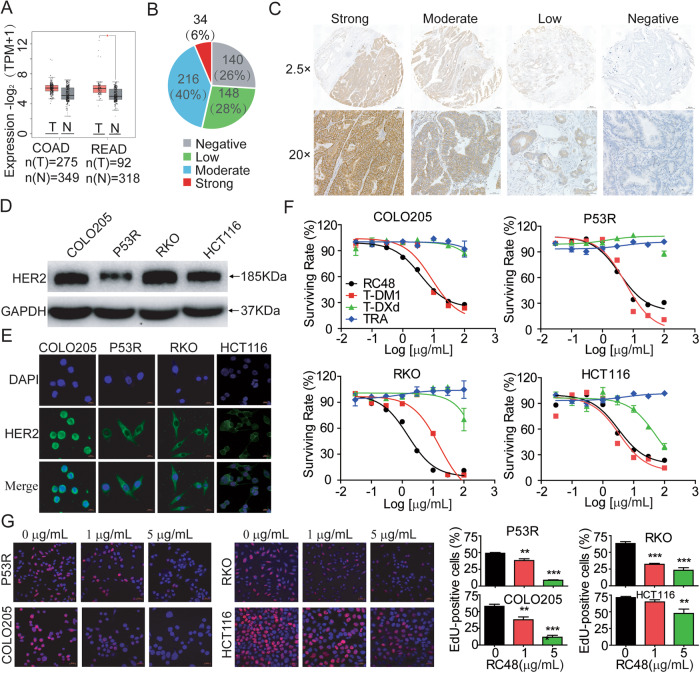
RC48 significantly inhibited cell proliferation in HER2-positive CRC cell lines. **A** DR5 mRNA overexpression in human CRC versus normal colon. Box plot has been downloaded from the GEPIA database. **B** Percent of 538 CRC tumors in each of the four staining intensity, including strong, moderate, low, and negative. **C** Representative pictures of the 4 staining intensity of HER2 IHC on human CRC tumor tissue microarray. **D** The expression of human HER2 in COLO205, P53R, RKO and HCT116 cells quantified by Western blotting. **E** Plasma membrane and intracellular subcellular localization of HER2 protein (green) by immunofluorescence in COLO205, P53R, RKO and HCT116 cells. Overlay with DAPI (blue) is depicted. **F** P53R, RKO, COLO205 and HCT116 cells were treated with RC48, T-DM1, T-DXd and trastuzumab for 72 h, respectively. The cell viability was detected by Cell Titer-Glo Cell Viability assay. **G** P53R, RKO, COLO205 and HCT116 cells were treated by 0, 1 and 5 μg/ml RC48 for 48 h, then cell proliferation was determined by the EdU assay. Fluorescence images were obtained and analyzed with a fluorescence microscope. Data are presented as means ± SEM of three independent experiments. Images captured at 400×magnification, respectively. Scale bars = 20 µm. Data represent the mean ± SEM of at least three independent experiments and statistical significance was assessed by unpaired T-test (*p < 0.05; **p < 0.01; ***p < 0.001).

We sought to characterize the HER2 expression in CRC cohort according to GEPIA database. HER2 was significantly overexpressed in CRC tissues compared with normal colorectal tissues (Fig. 1A). Consistently, our data showed that HER2 was positive in 74% (398/538) of 538 human CRC patient specimens using immunohistochemistry assay, and HER2 revealed strong, moderate, low, and negative staining intensities in 6% (34/538, 40% (216/538), 28% (148/538) and 26% (140/538) of specimens, respectively (Fig. 1B–C).

To screen for relevant CRC cell lines for in vitro and in vivo evaluation of RC48, we determined HER2 protein expressions in a panel of CRC cell lines by western blotting and immunofluorescence. Our results showed that HER2 was overexpressed and the subcellular localization was validated in P53R, RKO, COLO205 and HCT116 cell lines, and we found that HER2 was localized on the cell membrane or in the cytoplasm (Fig. 1D, E). We then selected 4 human CRC cell lines to evaluate the in vitro efficacy of FDA-approved ADCs targeted HER2.

We firstly compared in vitro cytotoxicity between RC48, T-DM1 and T-DXd in COLO205, P53R, RKO and HCT116 cells using the Cell Titer-Glo Cell Viability assay. Our results showed that RC48 significantly inhibited the growth of the four CRC cell lines in a dose-dependent manner. The IC50 values of COLO205, P53R, RKO and HCT116 cells were 3.791 μg/mL, 3.699 μg/mL, 1.549 μg/mL, and 3.236 μg/mL, respectively. T-DM1 also displayed antiproliferative activity in COLO205, P53R, RKO and HCT116 cells, with IC50 values of 8.784 μg/mL, 5.762 μg/mL, 43.15 μg/mL, and 3.406 μg/mL, respectively. However, T-DXd showed moderate antiproliferative activity only in HCT116 cells with IC50 values of 53.16 μg/mL but not COLO205, P53R and RKO cells, and trastuzumab did not show apparent cytotoxicity in these HER2-positive CRC cell lines (Fig. 1F). Therefore, RC48 monotherapy demonstrated more excellent antiproliferative activity in vitro among the three ADCs, and chose RC48 for further in vitro and in vivo evaluation.

To further assess the anti-proliferative effect of RC48, P53R, RKO, COLO205 and HCT116 cells cells were treated with RC48 via EdU staining assay. Our data revealed that RC48 significantly inhibited the proliferation in dose-dependent manner in P53R, RKO, COLO205 and HCT116 cells (Fig. 1G). Therefore, these results provide further evidence that RC48 could markedly suppress CRC cell proliferation in vitro.

### RC48 evidently triggered apoptosis and cell cycle arrest in vitro

**Fig. 2:**
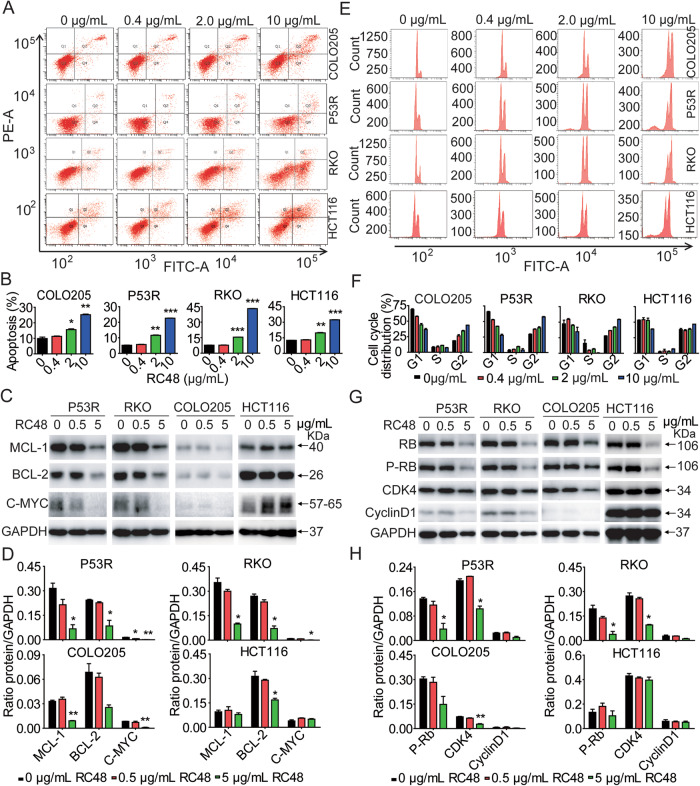
RC48 induced apoptosis and cell cycle arrest in HER2-positive CRC cells. P53R, RKO, COLO205 and HCT116 cells were treated with indicated concentrations of RC48 for 48 h, respectively. **A**, **B**, **E**, **F** The cells were stained with an anti-Annexin V-FITC antibody and PI for apoptosis analysis by flow cytometry. Annexin V-FITC-positive cells were defined as apoptotic (**A**, **B**). And the induction of cell cycle analysis in the CRC cells was detected by flow cytometry (**E**, **F**). The expression of critical molecules in the antiapoptotic (**C, D**) and cell cycle (**G, H**) pathway in CRC cells were examined by immunoblot analysis. Quantification was performed using Image J software. Data represent the mean ± SEM of at least three independent experiments and statistical significance was assessed by unpaired T-test (*p < 0.05; **p < 0.01; ***p < 0.001).

To investigate the underlying mechanism of action of RC48, we evaluated the impact of RC48 on cell cycle and apoptosis in P53R, RKO, COLO205 and HCT116 cells. Flow cytometry analysis was carried out to verify that cell apoptosis is involved in the cytotoxicity of RC48. After treatment with 0, 0.4, 2 and 10 μg/mL RC48 for 48 h, RC48 could markedly induce cell apoptosis in a dose-dependent manner in P53R, RKO, COLO205 and HCT116 cells (Fig. 2A, B). At the molecular level, RC48 alone significantly decreased the expression of the anti-apoptotic proteins such as BCL-2, MCL-1 and C-MYC in P53R, RKO and COLO205 cells, while the expression of MCL-1 and C-MYC were unchanged in HCT116 cells (Fig. 2C, D). In tumor cells, MYC expression is often elevated and no longer controlled by mitogenic signaling. The reason for this change is unknown. It is also unclear whether the deregulation or elevated expression of C-MYC is important for its role in cancer development. Interestingly, C-MYC can trigger apoptosis, especially under conditions of stress or lack of survival factors that often occur in tumors. However, it is still uncertain to what extent this apoptosis actually limits the cancer-causing abilities of C-MYC [31].

In addition, RC48 significantly induced cell cycle changes characterized by a decrease in G0-G1 phase, with a concomitant increase in G2-M phase in HER2-positive P53R, RKO and COLO205 cells (Fig. 2E, F). The dose-dependent cell cycle arrest was supported by decreased expression of CDK4 and p-Rb in P53R, RKO and COLO205 cells. However, expression of CDK4 and phosphorylation level of Rb were unchanged in HCT116 cells, and the expression Cyclin D1 wasn’t affected in P53R, RKO, COLO205 and HCT116 cells (Fig. 2G, H). Taken together, these findings displayed that RC48 partially inhibited tumor cell proliferation by inducing cell cycle arrest and apoptosis, ultimately leading to cell death.

### RC48 alone effectively induced CRC tumor regression in CDX and PDX Models

**Fig. 3:**
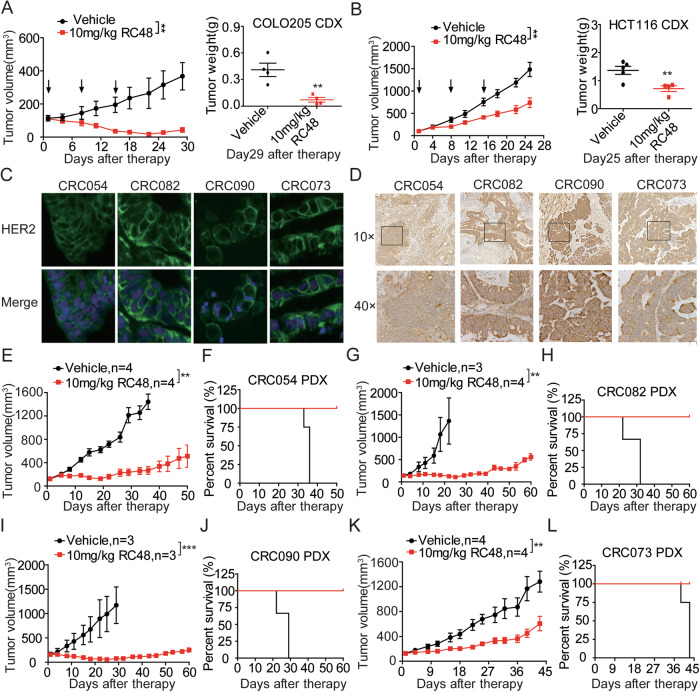
Antitumor activity of RC48 alone in mouse CRC CDX and PDX models. **A**, **B** Antitumor activity of RC48 in mouse COLO205 and HCT116 CDX models. BALB/c nude mice were injected s. c. with 5× 10^6^ COLO205 or HCT116 cells. Mice bearing xenografts approximately averaged 100–150 mm^3^ were intravenously received saline and 10.0 mg/kg of RC48. Tumor sizes and body weight were measured twice a week. Tumor growth curve and transplanted tumor weights were assessed at the end of the experiment in mouse COLO205 (**A**) and HCT116 (**B**) CDX models. Immunofluorescence analysis (**C**) and immunohistochemical (**D**) of HER2 expression in the transplanted tumors derived from the CRC054, CRC082, CRC090 and CRC073 PDX mice. Antitumor activity (**E**, **G**, **I**, **K**) and survival benefit (**F**, **H**, **J**, **L**) of RC48 in the CRC054 (**E**, **F**), CRC082 (**G**, **H**), CRC090 (**I**, **J**) and CRC073 (**K**, **L**) PDX models. RC48 and vehicle groups were intravenously administered. Immunofluorescence images captured at 630× magnification, scale bars = 10 µm. Immunohistochemical images captured at 100× and 400× magnification, scale bars = 100 and 20 µm, respectively.

We sought to explore the therapeutic effect of RC48 in vivo. In the COL205 and HCT-116 CDX models, mice were intravenously administered at 10 mg/kg RC48 for QW ×3 (once a week). RC48 significantly induced tumor regression, and terminal tumor growth inhibition (TGI) of 88.17% and 50.22% was observed in COL205 and HCT-116 CDX models, respectively (Fig. 3A, B). Furthermore, none of the treatments resulted in significant changes in mouse body weight of these CDX models (Supplemental Fig. 1A, B).

Patient-derived xenografts (PDXs) are generally considered to be one of the most references for evaluating in vivo antitumor efficacy and have been widely used in preclinical drug development [32–35]. In order to more effectively confirm the efficacy of RC48 in clinical indications, the antitumor activity of RC48 on 4 PDX models of CRC were performed. We first determined HER2 protein expressions in 4 CRC PDX models by immunofluorescence and immunohistochemical assay. Our results demonstrated that HER2 was overexpressed and the subcellular localization was validated in 4 CRC PDX models (Fig. 3C, D). In these PDXs, RC48 at 10 mg/kg displayed significantly potent tumor inhibitory effects compared with the vehicle groups (Fig. 3E, G, I, K). And RC48 resulted in a significant improvement in overall survival compared with their corresponding vehicle groups (Fig. 3F, H, J, L). Furthermore, none of the treatments resulted in significant changes in mouse body weight of these PDX models (Supplementary Fig. 1C–F). Altogether, RC48 alone exhibits superior antitumor effects in CRC CDX and PDX models and can be used as a drug candidate for the treatment of CRC.

### RC48 synergized with GEM significantly enhanced antiproliferative activity in vitro

Currently, chemotherapy combined with targeted therapy is still the main treatment for advanced CRC. Of note, combinational therapy of ADC and other anticancer drugs has currently become an important direction in the development of ADC drugs. Thus, to explore the combinational therapeutic efficacy of RC48, we first screened some first-line anticancer drugs in P53R, RKO, HCT116 and COLO205 cells using Cell Titer-Glo cytotoxicity assays. Our data demonstrated that GEM exhibited stronger antiproliferative activity compared to the other first-line anti-tumor agents. The IC50 values of GEM for P53R, RKO, HCT116 and COLO205 cells were 11.37 nM, 79.24 nM, 6.951 nM, and 1.692 μM, respectively (Supplementary Fig. 2). GEM is a FDA-approved deoxycytidine analog that is now widely used as an anti-cancer chemotherapeutic drug [24].

**Fig. 4:**
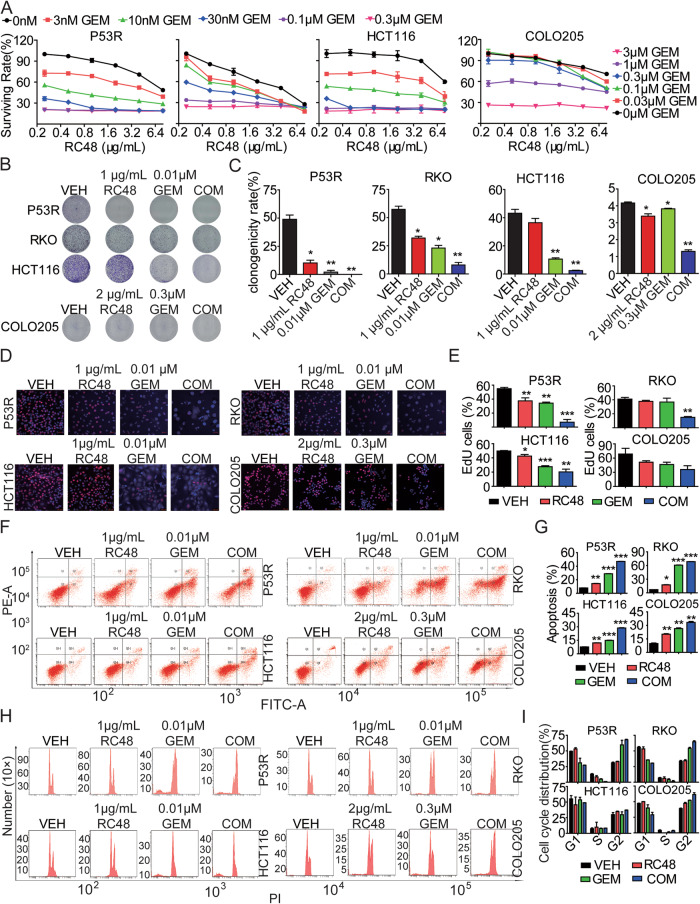
The synergistic antitumor effects of RC48 and/or GEM on HER2-positive CRC cells. **A** Cytotoxicity of RC48 combined with GEM in P53R, RKO, HCT116 and COLO205 cells were determined by CellTiter-Glo® cytotoxicity assays. The dose of RC48 monotherapy was set as 0, 0.25, 0.5, 1, 2, 4 and 8 μg/mL in P53R, RKO, HCT116 and COLO205 cells, while GEM was set as 0, 3, 10, 30, 100 and 300 nM in P53R, RKO and HCT116 cells, and GEM was set as 0, 0.03, 0.1, 0.3, 1 and 3 μM in COLO205 cells. **B**, **C** P53R, RKO, HCT116 and COLO205 cells were treated with RC48, GEM and their COM for 14 days. The proliferative ability of CRC cells was investigated via colony formation assays. **D**, **E** P53R, RKO, HCT116 and COLO205 cells were treated with RC48, GEM, or their COM for 48 h, then cell proliferation was determined by EdU assay. **F**, **G** P53R, RKO, HCT116 and COLO205 cells were treated with RC48, GEM, or their COM for 48 h. The cells were stained with an anti-Annexin V-FITC antibody and PI for apoptosis analysis by flow cytometry. Annexin V-FITC-positive cells were defined as apoptotic. **H**, **I** P53R, RKO, HCT116 and COLO205 cells were treated with RC48, GEM, or their COM for 48 h.The induction of cell cycle analysis in P53R, RKO, HCT116 and COLO205 cells was detected by flow cytometry. Data represent the mean ± SEM of at least three independent experiments and statistical significance was assessed by unpaired T-test (*p < 0.05; **p < 0.01; ***p < 0.001).

We thus further evaluated the synergistic effect upon treatments with RC48 plus GEM in P53R, RKO, HCT116 and COLO205 cells by Cell TiterGlo Cell Viability assay. The dose of RC48 monotherapy was set as 0, 0.25, 0.5, 1, 2, 4 and 8 μg/mL in P53R, RKO, HCT116 and COLO205 cells, while GEM was set as 0, 3, 10, 30, 100 and 300 nM in P53R, RKO and HCT116 cells, and GEM was set as 0, 0.03, 0.1, 0.3, 1 and 3 μM in COLO205 cells. Several combinational doses showed synergistic effects in P53R, RKO, HCT116 and COLO205 cells. Among them, we chose 1 μg/mL RC48 and 0.01 μM GEM as the candidate combination dose in P53R, RKO and HCT116 cells, while 2 μg/mL RC48 and 0.3 μM GEM as the candidate combination dose in COLO205 cells, to perform the subsequent in vitro experiments (Fig. 4A). Furthermore, RC48 combined with GEM significantly inhibited proliferation in P53R, RKO, HCT116 and COLO205 cells colony formation assay (Fig. 4B, C) and EdU assay (Fig. 4D, E).

We further evaluated the impact of combined therapy on cell cycle and apoptosis by flow cytometry in P53R, RKO, HCT116 and COLO205 cells. Combination of RC48 and GEM cooperatively induced a significant increase in the percentage of apoptotic cells compared with the percentage observed for RC48 or GEM alone in P53R, RKO, HCT116 and COLO205 cells (Fig. 4F, G). In addition, combination of RC48 and GEM significantly induced cell cycle changes characterized by a decrease in G0-G1 phase, with a concomitant increase in G2-M phase in P53R, RKO and COLO205 cells, but combinational treatment had no effect on cell cycle in HCT116 (Fig. 4H, I). Collectively, these results demonstrated that the cell growth inhibition observed with the combined therapy could be mechanistically explained at least in part involved the induction of apoptosis and cell-cycle arrest.

### Synergistic effects of RC48 plus GEM on global gene expression profiling in CRC cells

**Fig. 5:**
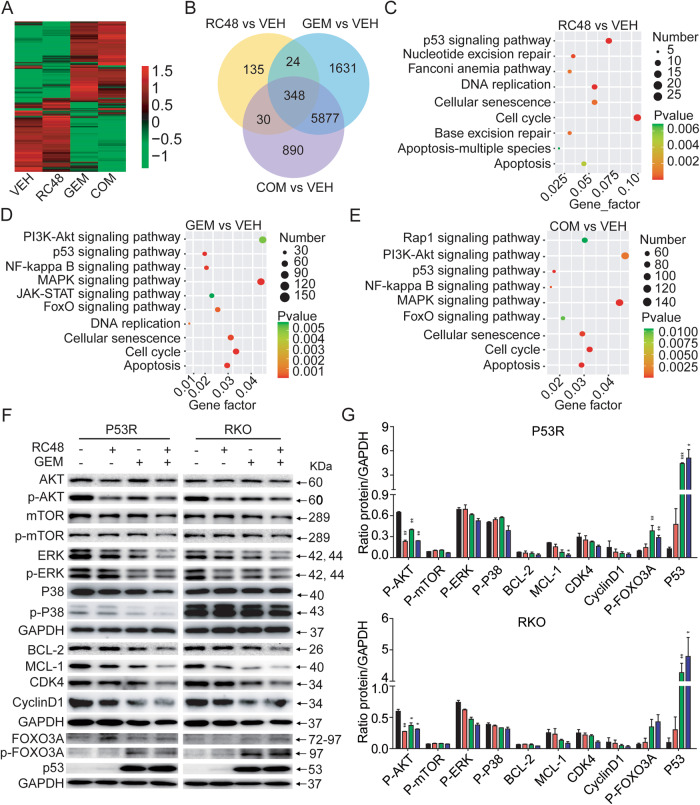
Synergic effects of RC48 and GEM combinational treatment on global gene expression profile in CRC cells. **A** All DEGs in the vehicle-treated group (VEH; *N* = 3), 1 μg/mL RC48 (*N* = 3), 0.01 μM GEM (*N* = 3) and RC48 plus GEM (COM; *N* = 3) treated groups in P53R cell. **B** Numbers of overlapped DEGs in the RC48, GEM, and COM treated samples as compared with the VEH group in P53R cell. KEGG analysis of DEGs in the RC48 (**C**), GEM (**D**) and COM (**E**) treated samples compared with the VEH-treated samples in P53R cells. **F**, **G** Analysis of the PI3K/AKT pathway, AMPK pathway, p53 pathway, FOXO pathway, cell cycle-related proteins and antiapoptotic proteins in P53R and RKO cells. Quantification was performed using Image J software. GAPDH was used as the loading control.

Based on above data in Fig. 4, combination therapy of RC48 and GEM showed more superior antitumor activity in P53R and RKO cells compared with in COLO205 and HCT116 cells. We thus further comprehensively unravel the potential mechanisms underlying the synergistic effects in P53R and RKO cells by bulk RNA-seq analysis of RC48, either alone or in combination with GEM. We analyzed the differentially expressed genes (DEGs) in each group using multidimensional scaling (Fig. 5A and Supplemental Fig. 3A). In these cells, a significant number of genes exhibited differential expression in the combination group compared to the vehicle group. Specifically, in the combination group, 7145 (26.16%) and 3873 (14.51%) DEGs were shown in P53R, and RKO cells, respectively. Among these genes, transcripts of 3877 (14.23%) and 2229 (8.35%) were upregulated, while transcripts of 3258 (11.93%) and 1644 (6.16%) were downregulated compared to the vehicle group (Supplemental Fig. 4). For DEGs, RC48 treatment (*N* = 537) and combinational treatment group (*N* = 7145) decreased fewer genes than GEM group (*N* = 7880) in P53R cell. Consistently, DEGs of RC48 (*N* = 1380) and combinational treatment group (*N* = 3873) was fewer than in the GEM treatment group (*N* = 6232) in RKO cell(Fig. 5B and Supplementary Fig. 3B). It suggested that RC48 alone had a limited effect on the overall gene expression program, while it was synergistic when combined with GEM.

The KEGG analysis of the DEGs showed that multiple pathways were regulated by RC48 treatment in P53R and RKO cells, such as p53 signaling pathway, apoptosis, DNA replicaiton, etc. Whereas GEM primarily modulated the PI3K-AKT pathway, MAPK pathway, p53 signaling pathway, Foxo pathway, apoptosis, cell senescence and cell cycle, etc. Interestingly, as with GEM treatment group, the combinational treatment group also clustered in these pathways (Fig. 5C–E, Supplementary Figs. 3C–E and 5). We thus further validated the combinational effects of RC48 and GEM on biological processes and related signaling pathway mentioned above in P53R and RKO cells. Consistent with the RNA-Seq analysis, we found that compared with RC48 or GEM alone, the combination of RC48 and GEM did not further synergically inhibit the phosphorylation of AKT and mTOR, while RC48 alone decreased the phosphorylation of AKT. This may be due to the fact that GEM does not inhibit the downstream signaling pathway of HER2. Meanwhile, the combination of RC48 and GEM decreased the phosphorylation of ERK, and increased the phosphorylation of FOXO3A compared with RC48 or GEM alone. These results suggested that RC48 plus GEM inhibit these pathways in P53R and RKO cells. In P53R and RKO cells, the combination therapy also confirmed downregulation of cell cycle-related proteins (CDK4 and cyclinD1). In addition, the levels of apoptosis proteins MCL-1 in P53R and RKO cells were decreased in the combined treatment group, and the levels of senescence marker p53 were also up-regulated (Fig. 5F, G).

Collectively, these data clearly demonstrated that RC48 plus GEM regulated multiple signaling pathways, such as PI3K-AKT pathway, MAPK pathway, p53 signaling pathway, Foxo pathway, apoptosis, cell senescence and cell cycle, etc, to exert its antitumor activity in CRC cells.

### Combined treatment of RC48 and GEM significantly affected unique CRC-associated prognostic genes

**Fig. 6:**
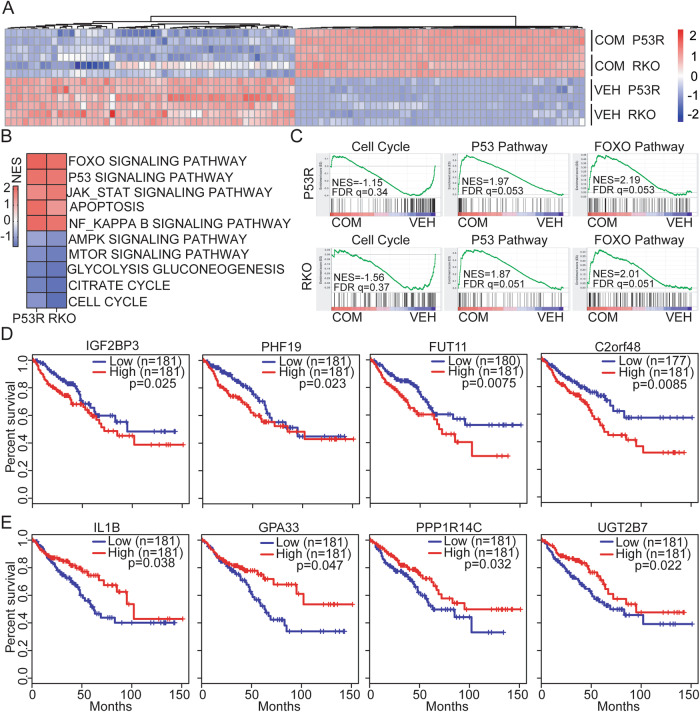
RC48 plus GEM treatment affected unique CRC-associated prognostic genes. **A** Heatmap showing the mRNA levels of the top 100 genes (50 up or downregulated) after RC48 plus GEM combinational treatment (COM). The expression levels of each gene were normalized to the total mRNA abundance of each sample and compared with that of vehicle-treated group (VEH). **B** The top ranked positively and negatively enriched gene sets identified using GSEA in response to combinational treatment. GSEA was conducted with top common DEGs in P53R and RKO cells after combinational treatment using 50 HALLMARK gene sets database in MSigDB. **C** GSEA plots showing strong negative enrichment of the cell cycle, and positively enrichment of the P53 and FOXO pathway in P53R and RKO cells in response to combinational treatment. NES Normalized Enrichment Score, FDR false discovery rate. **D**, **E** Kaplan–Meier estimate of overall survival based on expression of DEGs (TCGA CRC Cohort). Relative high expression of IGF2BP3, PHF19, FUT11 and C2orf48 genes (**D**), and relative low expression of IL1B, GPA33, PPP1R14C and UGT2B7 (**E**) were associated with poor overall survival in CRC cohort. Log rank (Mantel–Cox) test was used for significance; *p* < 0.05 was considered significant.

Next, Gene Set Enrichment Analysis (GSEA) was conducted using the 50 Hallmark gene set collections in MSigDB for the identification of specifically enriched biological pathways following combinational therapy of RC48 and GEM. GSEA analysis of the common DEGs showed strong negative enrichment for the gene sets involved in the cell cycle in P53R and RKO cells treated with combinational treatment, and strong positive enrichment of gene sets involved in p53 signaling pathway and Foxo pathway (Fig. 6A–C). The upregulated and downregulated gene sets are presented in Fig. 6B and Supplementary Table 2.

To validate the biologic relevance of the genes in CRC regulated by RC48 plus GEM, we explored the TCGA dataset from the CRC cohort to confirm whether any of the top 200 (100 up and down) DEGs identified by RNA-Seq analysis were aberrantly expressed and/or associated with outcome. Four downregulated DEGs (IGF2BP3, PHF19, FUT11 and C2orf48) and four upregulated DEGs (IL1B, GPA33, PPP1R14C and UGT2B7) were observed in P53R and RKO cells following combinational therapy, and IGF2BP3, PHF19, C2orf48, IL1B, GPA33, PPP1R14C and UGT2B7 were overexpressed in CRC TCGA cohort (Supplementary Fig. 6). Furthermore, Kaplan-Meier survival analysis indicated that high or low expression of each of these genes was associated with poor overall survival in CRC cohort (*p* < 0.05) (Fig. 6D, E).

### Combination therapy with RC48 and GEM in CDXs and PDXs

**Fig. 7:**
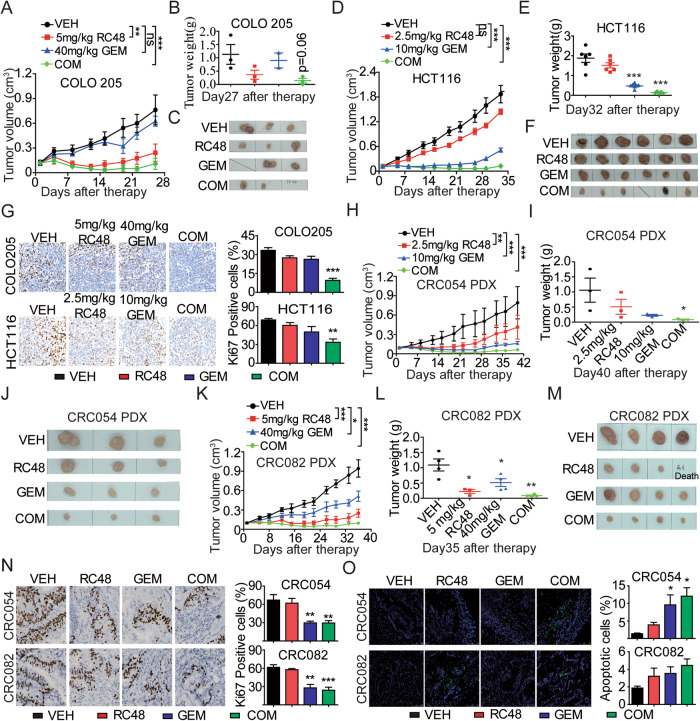
RC48 plus GEM synergistically inhibited CRC tumor growth in mouse CDX and PDX models. Tumor growth curve, transplanted tumor weight, and images are displayed in COLO205 (**A−C**) and HCT116 (**D−F**) CDX models. **G** Images of immunohistochemical staining for Ki67 in COLO205 and HCT116 CDX mouse xenograft tumors. Tumor growth curve, transplanted tumor weight, and images were evaluated in CRC054 (**H**–**M**) and CRC082 (**K**–**M**) PDX models. **N** Images of immunohistochemical staining for Ki67 in CRC054 and CRC082 PDX mice xenograft tumors. **O** Images of immunofluorescence TUNEL of CRC054 and CRC082 PDX mouse xenograft tumors. RC48 and vehicle groups were intravenously administered and GEM groups were intraperitoneally administered. IHC Images captured at 400×magnification, Scale bars = 20 µm. TUNEL Images captured at 200×magnification, respectively. Scale bars = 500 µm. And statistical significance was assessed by unpaired T-test (*p < 0.05; **p < 0.01; ***p < 0.001).

To explore whether our in vitro findings could be translated in an in vivo setting, the tumoricidal activity of RC48 combined with GEM was further investigated in CDXs and PDXs. First, in BALB/c nude mice bearing subcutaneous COLO205 and HCT116 CDX models, combination of RC48 and GEM resulted in a significant antitumor effect compared with all other treatment groups, and TGI of 85.81% and 93.43% was observed, respectively (Fig. 7A–F). In contrast, RC48 at 5 mg/kg exerted moderate antitumor effects while 40 mg/kg GEM had no inhibitory effect on tumor growth in COLO205 CDX model (Fig. 7A–C). Similarly, 10 mg/kg GEM exerted moderate tumoricidal activity but 2.5 mg/kg RC48 had no inhibitory effect on tumor growth in HCT116 CDX model (Fig. 7D–F). In addition, all the CDX xenograft tumors were collected and prepared for molecular pathological analysis. IHC staining displayed that the Ki-67 expression in the combination group of RC48 and GEM was decreased compared with that in the monotherapy groups, indicating the antiproliferative activity of the combination treatment (Fig. 7G).

To further investigate the antitumor activity of combination therapy of RC48 and GEM in vivo, CRC054 and CRC082 PDXs were selected and established. First, mice bearing subcutaneous CRC054 xenografts were administrated with RC48 and/or GEM once a week for 3 times (QW×3) and the tumor growth was measured for 40 days. Comparing with the vehicle group, mice treated with 2.5 mg/kg RC48 or 10 mg/kg GEM group exhibited an obviously decreased tumor volume and the TGI of 47.82% or 79.80% were observed, respectively. Notably, combination of RC48 and GEM significantly induced antitumor activity compared with another monotherapy groups, and the TGI was 92.15% (Fig. 7H–J). In the other CRC082 PDX model, comparing with the vehicle group, mice treated with 5 mg/kg RC48 or 40 mg/kg GEM group displayed a remarkably decreased tumor volume and the TGI of 78.78% or 51.23% were observed, respectively. Of note, the combination between 5 mg/kg RC48 and 40 mg/kg GEM had a significantly higher antitumor activity comparing with another monotherapy groups, and the TGI was 89.96% (Fig. 7K–M). In addition, all the PDX xenograft tumors were collected and prepared for molecular pathological analysis. IHC staining displayed that a decreased expression level of Ki67 in the xenograft tumor tissues in the combination group of RC48 and GEM was observed compared with that in the monotherapy groups, indicating the antiproliferative activity of the combination treatment (Fig. 7N). Similarly, immunofluorescence images showed an intense terminal deoxynucleotidyl transferase-mediated dUTP-biotin nick end labeling (TUNEL) signal in the combination group of RC48 and GEM (Fig. 7O).

More importantly, this combination treatment appeared to be well-tolerated in these CDX and PDX models, as no significant body weight changes were observed in these animals during the experimental process (Supplementary Fig. 7). To further evaluate changes in the organs of mouse xenograft models in response to RC48 and/or GEM, the organs of the CDX and PDX model mice were stained by H&E assay to investigate the in vivo toxicity of all the treatment groups. Compared with the control group, combination of RC48 and GEM demonstrated no obvious toxic side-effects on heart, liver, spleen, lung and kidney, suggesting this combination therapy has negligible toxicity in vivo (Supplementalry Fig. 8).

Collectively, consistent with the data from the in vitro experiments, these findings from the in vivo experiments support the superior tumoricidal effect of the combinational therapy, further providing initial rational for subsequent clinical development of the combination of RC48 and GEM as a novel therapeutic strategy for CRC patients.

## Discussion

HER2 has become one of the most successful therapeutic target for the treatment of various malignant tumors, including breast cancer, gastric cancer, esophageal cancer, head and neck tumors and urothelial cancer, etc. ADC have recently become one of the most popular research fields for anti-tumor agents, thereby representing the development direction of personalized precision therapy. It is an important topics that Optimizing the efficacy and safety of ADCs by systematic preclinical evaluation of mono- or combinational therapy for ADC development. Many patients have greatly benefited from these ADCs in mono- or combinational therapy [36]. Thus, it is very important to explore ADC targeting HER2 alone and combination treatment of HER2-positive advanced CRC.

Here, we characterized HER2 expression in human CRC patient specimen, cell lines and PDX xenograft tumors as a biomarker for responses to RC48. Our data demonstrated that these CRCs could highly express HER2 and localized at the plasma membrane, consistent with previous studies on the expression of HER2 in CRC [6, 37–39]. We thus went on to investigate the tumoricidal effects and the underlying mechanism of RC48 in both in vitro and in vivo preclinical models of CRCs. We displayed that RC48 has antitumor activity by inducing cell cycle arrest and apoptosis in HER2-positive CRC cell lines. Of note, these data from the in vivo experiments support the potential antitumor activity of RC48. Consistent with previous reports [20–22], RC48 had superior antitumor activity in a dose-dependent manner on gastric cancer /breast carcinoma cell lines, CDX and PDX models with HER2 expression. It has been reported that the antitumor activity of T-DXd is determined by the expression of HER2 protein, not its gene amplification status [40]. In addition, while high level of HER2 expression is positively correlated with the efficacy of monoclonal antibody-based therapies, it is not necessary for HER2-targeting ADCs due to the unique mechanism of ADC-mediated cytotoxicity. For example, Modi et al. showed that trastuzumab deruxtecan (T-DXd) resulted in significantly longer progression-free and overall survival in patients with HER2-low metastatic breast cancer in the DESTINY-Breast04 Clinical Trials (gov number, NCT03734029) [41]. Therefore, we selected CRC PDXs based whether they express the HER2 protein, as determined by IHC, regardless of the expression level. Similar to previous reports, we found the level of HER2 expression did not correlate with the efficacy of RC48-ADC in CRC PDX models, in line with the notion that HER2 expression level as measured by IHC is not a reliable predicative biomarker for HER2-targeting ADCs and can not be used for patient stratification.

Combination therapy, in which some drugs with different mechanisms are co-administered, is widely used in biomedical research and clinical application. Chemotherapy combined with targeted therapy is still the main treatment for advanced CRC. Of note, combinational therapy of ADC and other anticancer drugs has currently become an important direction in the development of ADC drugs [42, 43]. For example, PARP inhibitors enlarged the antitumor activity of sacituzumab govitecan in triple-negative breast cancer [44] and BCL-2/XL inhibitors Enhances the cytotoxicity of T-DM1 [45]. We thus combined RC48 with FDA-approved first-line chemotherapy drugs for the treatment of advanced CRC. Here, our data showed that combinational therapy of RC48 and gemcitabine had the synergistic antitumor effect in CRC cell lines. And the in vivo results confirmed that combinational treatment had a significantly better antitumor effect in the CRC CDX and PDX models compared with GEM or RC48 alone. This is in agreement with the synergistic effects that we observed between RC48 and GEM in vitro. CRC is a highly heterogeneous disease, exhibiting variable survival outcomes and therapeutic vulnerabilities. Therefore, we observed different treatment outcomes and survivals in CDX and PDX models treated with the drug combination. In COLO205 CDX model, complete remission (CR) was observed in one of three animals from day 15 to day 27, while the other two mice showed partial relapse from day 19 to the end of the experiment on day 27. In comparison, the ADC combination showed superior and sustained anti-tumor activity in HCT116 CDX, CRC052 PDX and CRC082 PDX up to the end of the experiment on day 32, day 40 and Day 35, respectively. However, based on current observations, it is likely that this ADC combination would be able to maintain a long-term efficacy in most CRC models should the dosing regimen continuous. Thus, optimizing the strategy of the combinations of chemotherapy drugs with ADCs, targeted molecules or immunotherapies for the treatment of advanced CRC patients, are still being explored.

To define the mechanism underlying the observed antitumor activity of RC48, we investigated their effects on CRC cell cycle and apoptosis. RC48 significantly decreased the expression level of the apoptotic proteins (BCL-2, MCL-1, C-MYC) and cell cycle-related proteins (CDK4, Cyclin D1, and p-Rb) in P53R, RKO and COLO205 cells, while these proteins were unchanged in HCT116 cells. In agreement with previous findings about the ADC based on T-DM1 and Rituximab-MMAE, apoptosis and cell cycle arrest were triggered by RC48 [46–48]. Thus, these data suggested that antitumor effects observed with RC48 treatment could be mechanistically explained at least in part involved the induction of cell-cycle arrest and apoptosis in CRC cells.

Moreover, bulk RNA-seq analysis further revealed that combinational therapy primarily affected DEGs related to PI3K-AKT pathway, MAPK pathway, p53 signaling pathway, Foxo pathway, apoptosis, cell senescence and cell cycle, etc. Consistent with the RNA-seq analysis, combinational treatment decreased the phosphorylation of ERK, and increased the phosphorylation of FOXO3A, indicating RC48 plus gemcitabine inhibited these pathways in P53R and RKO cells. Also, the down-regulation of cell cycle-related proteins (CDK4 and cyclinD1) and apoptotic protein (MCL-1), and up-regulation of senescence marker (p53) in the combinational treatment were confirmed in P53R and RKO cells. Thus, these above results demonstrated that RC48, either alone or in combination with GEM, inhibited tumor cell proliferation by regulating many signaling pathways, ultimately leading to cell death.

Additionally, global gene expression profile and gene set enrichment analysis identified several important new gene sets that were regulated by combinational therapy in P53R and RKO cells, strong negative enrichment for the gene sets involved in cell cycle progression, and strong positive enrichment of gene sets involved in p53 signaling pathway and Foxo pathway. Intriguingly, the high or low expression level of eight genes (IGF2BP3, PHF19, FUT11, C2orf48, IL1B, GPA33, PPP1R14C and UGT2B7) were associated with poor prognosis in CRC cohort. Of note, it has been reported that these genes were involved in tumor cell growth, proliferation, invasion and metastasis in various cancers [49–53].

Of course, we had some limitations in our study. Most data was only dependent on the expression of HER2 protein, but these CRC PDX models lacked tumor profiling analysis by genome sequencing or whole-exome sequencing. Moreover, the DEGs related to overexpression and poor prognosis are still needed to be further explored.

In conclusion, our findings highlight RC48 alone or combinational therapy, as an effective therapeutic method for patients with CRC. It will be valuable reference to investigate the efficacy of RC48 in combination with chemotherapy drugs, targeted molecules or immunotherapies in HER2-positive solid tumors, facilitating entry into the clinical stage as a promising therapeutic strategy.

### Supplementary information

Original Data File

Supplementary information

## Data Availability

All data in our study are available upon reasonable request.
