# Supplementary figures and images for: Synergistic antitumor activity between HER2 antibody-drug conjugate and chemotherapy for treating advanced colorectal cancer
Source: Cell Death Dis. 2024 Mar 5;15(3):187. doi: 10.1038/s41419-024-06572-2 (PMC10914798; doi:10.1038/s41419-024-06572-2)

# Uncropped scans of blots

Figure 1A

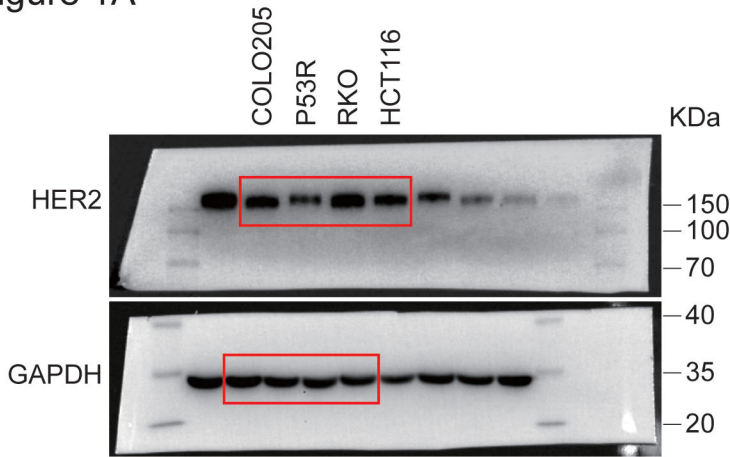

Figure 2C

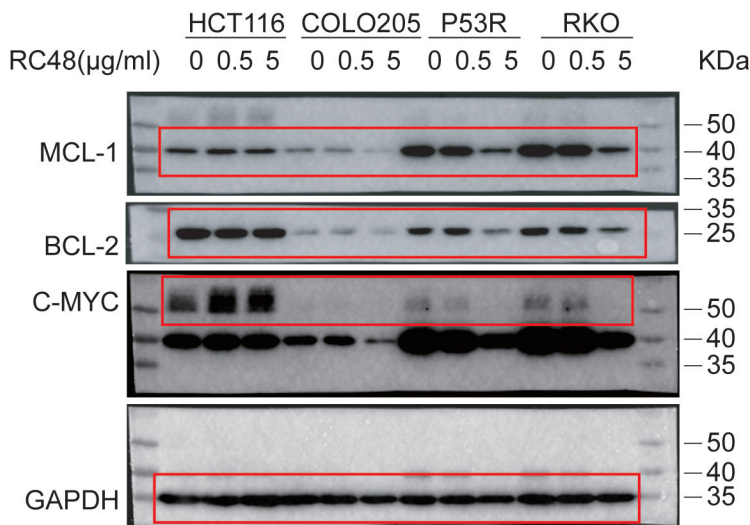

Figure 2G

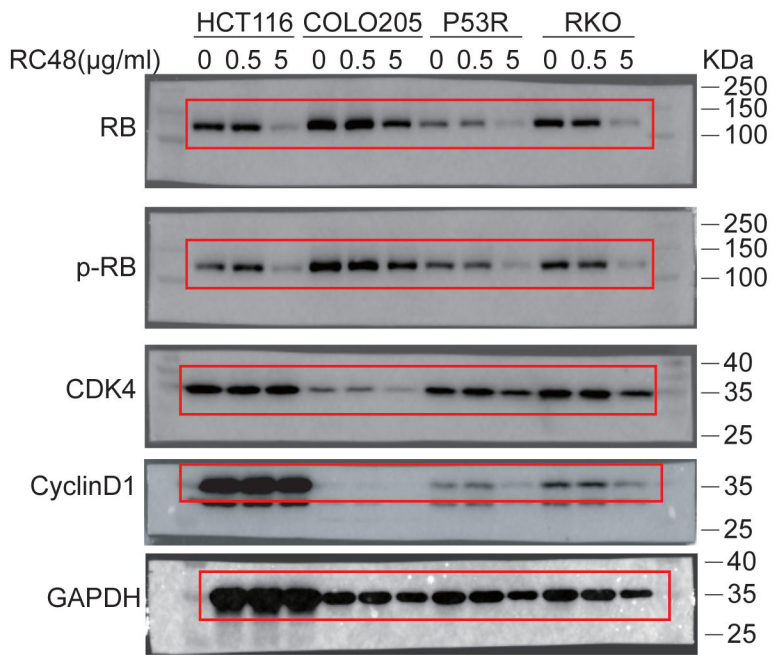

Figure 5F

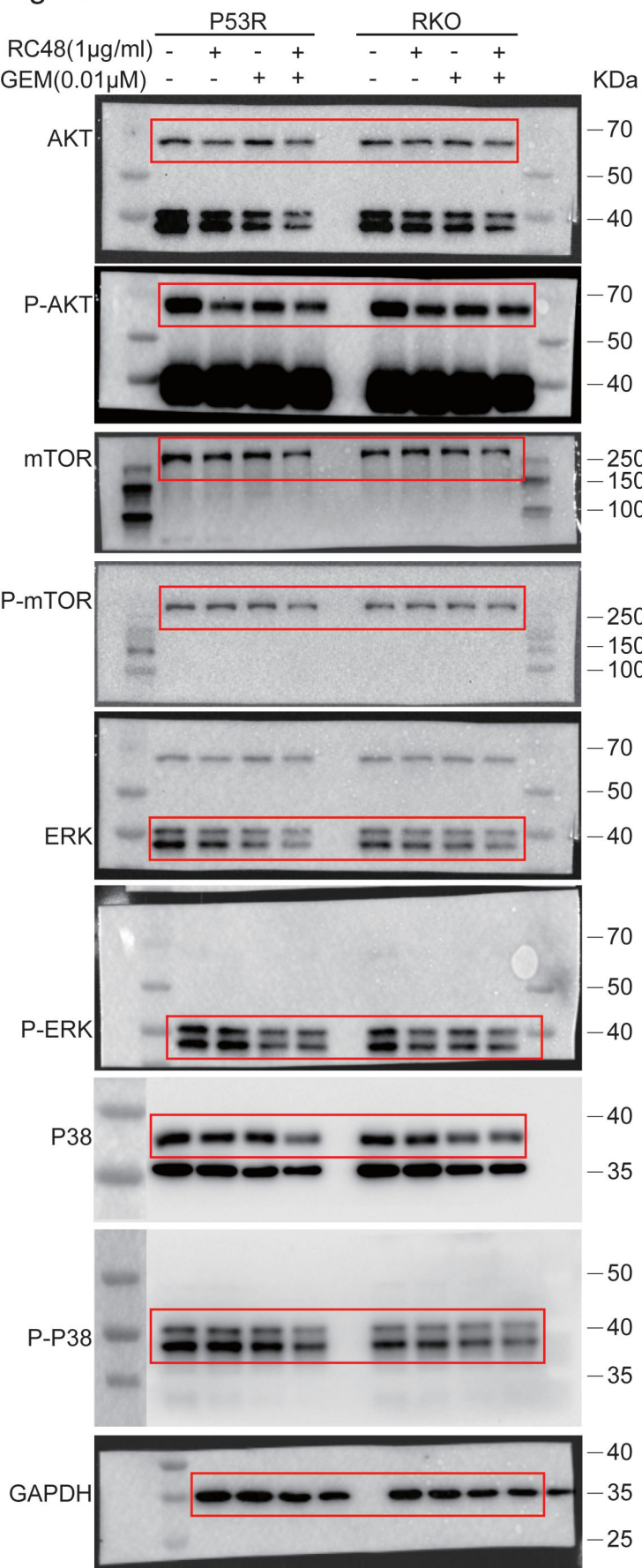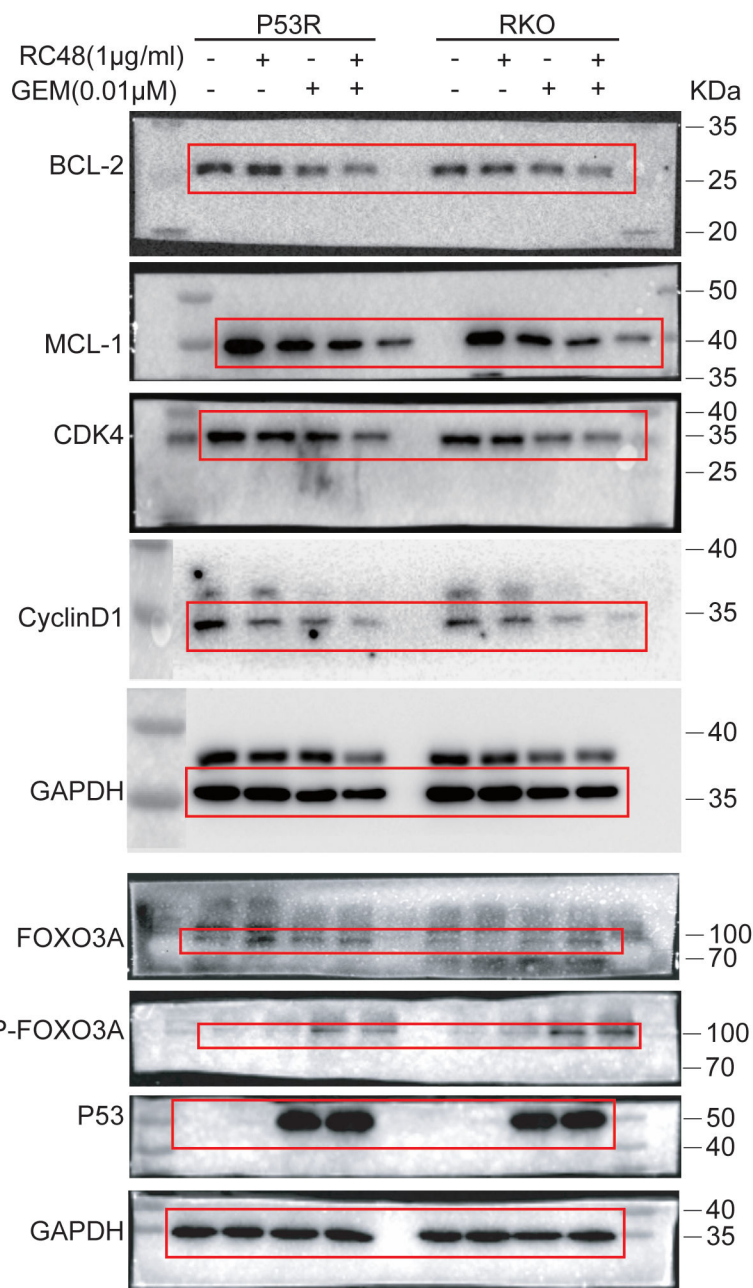

Supplement: Supplementary file 1 — Original Data File [file 41419_2024_6572_MOESM1_ESM.pdf]
