# Supplementary material for: Synergistic antitumor activity between HER2 antibody-drug conjugate and chemotherapy for treating advanced colorectal cancer
Source: Cell Death Dis. 2024 Mar 5;15(3):187. doi: 10.1038/s41419-024-06572-2 (PMC10914798; doi:10.1038/s41419-024-06572-2)
Supplement: Supplementary file 2 — Supplementary information [file 41419_2024_6572_MOESM2_ESM.docx]

**Supplemental information**

#

# Supplemental Table 1. List of antibodies used for immunofluorescence and immunohistochemistry and western blot analysis

| Target | Company | Cat No. | Application | Dilution |
| --- | --- | --- | --- | --- |
| HER2 | Proteintech | 18299-1-AP | IHC/IF | 1：500 |
| Ki67 | CST | 9027 | IHC | 1：500 |
| AKT | CST | 9272 | WB | 1：2000 |
| p-Akt | CST | 4060 | WB | 1：2000 |
| mTOR | proteintech | 28273-1-AP | WB | 1：2000 |
| P-mTOR | CST | 2971 | WB | 1：1000 |
| p38 | CST | 8690 | WB | 1：2000 |
| p-p38 | CST | 4511 | WB | 1：2000 |
| ERK1/2 | CST | 4695 | WB | 1：2000 |
| p-ERK1/2 | CST | 4370 | WB | 1：2000 |
| C-MYC | CST | 18583 | WB | 1：2000 |
| FOXO3A | proteintech | 10849-1-AP | WB | 1：1000 |
| p-FOXO3A | Affinity | AF3020 | WB | 1：1000 |
| CDK4 | proteintech | 11026-1-AP | WB | 1：2000 |
| Cyclin D1 | proteintech | 26939-1-AP | WB | 1：1000 |
| RB | Affinity | AF6103 | WB | 1：2000 |
| p-RB | Affinity | AF3103 | WB | 1：2000 |
| Bcl-2 | Affinity | AF6139 | WB | 1：1000 |
| Mcl-1 | CST | 94296 | WB | 1：2000 |
| P53 | proteintech | 10442-1-AP | WB | 1：1000 |
| GAPDH | CST | 5174 | WB | 1：10000 |
| HRP-conjugated Affinipure Goat Anti-Rabbit IgG (H+L) | Proteintech | SA00001-2 | WB | 1：10000 |
| HRP-conjugated Affinipure Goat Anti-Mouse IgG (H+L) | Proteintech | SA00001-1 | WB | 1：10000 |

# Supplemental Table 2. List of GSEA analysis

| Gene set name | NES | NES | FDR q VALUE | FDR q VALUE |
| --- | --- | --- | --- | --- |
|  | P53R | RKO | P53R | RKO |
| FOXO SIGNALING PATHWAY | 2.19 | 2.01 | 0.053 | 0.051 |
| P53 SIGNALING PATHWAY | 1.97 | 1.87 | 0.053 | 0.051 |
| JAK_STAT SIGNALING PATHWAY | 1.61 | 1.84 | 0.059 | 0.055 |
| APOPTOSIS | 2.06 | 1.42 | 0.053 | 0.159 |
| NF_KAPPA B SIGNALING PATHWAY | 2.08 | 1.96 | 0.053 | 0.051 |
| AMPK SIGNALING PATHWAY | -0.95 | -1.17 | 0.497 | 0.496 |
| MTOR SIGNALING PATHWAY | -1.21 | -1.39 | 0.332 | 0.252 |
| GLYCOLYSIS _GLUCONEOGENESIS | -1.31 | -1.48 | 0.345 | 0.194 |
| CITRATE CYCLE | -1.34 | -1.54 | 0.385 | 0.242 |
| CELL CYCLE | -1.15 | -1.56 | 0.340 | 0.376 |


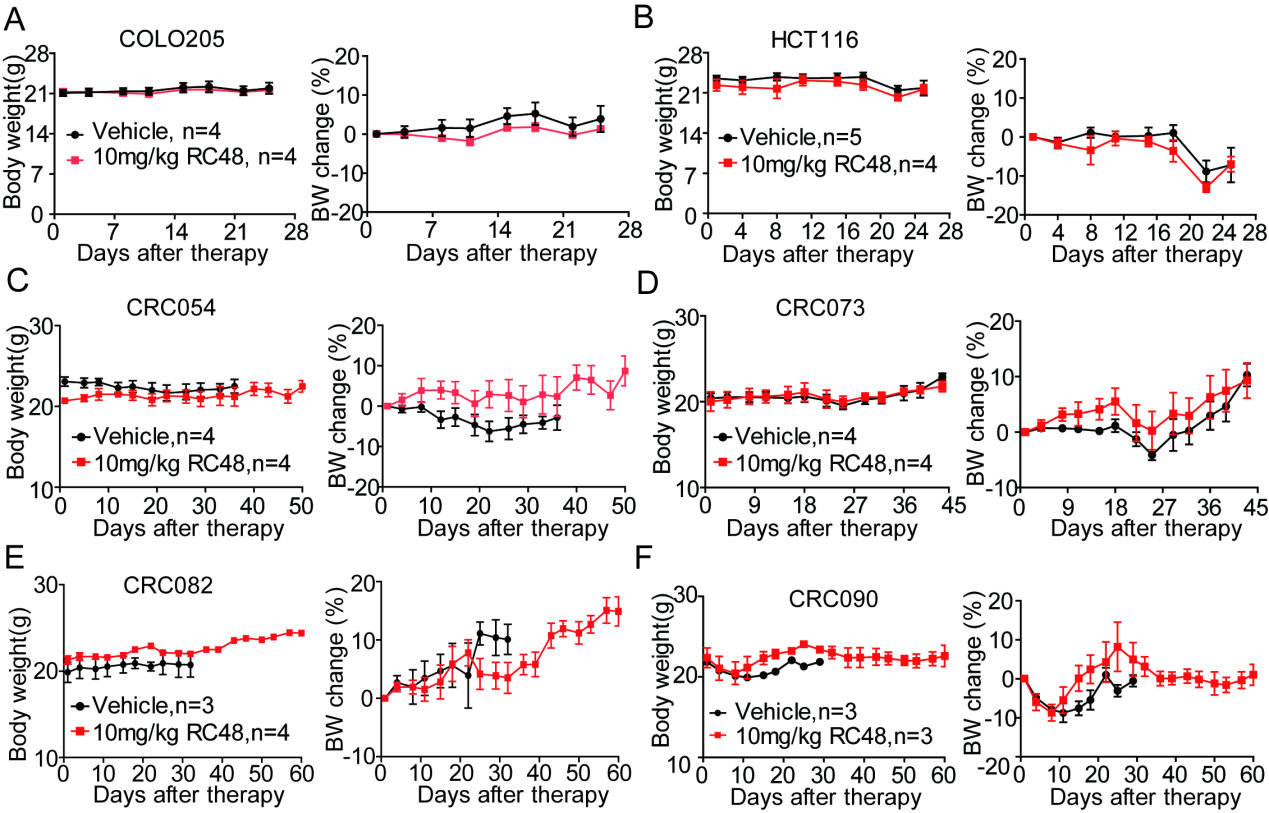


**Supplemental Figure 1.** Mice body weights and the changes of the body weights were assessed once every week for three times in COLO205 (A) and HCT116 (B) CDX models, and CRC054 (C), CRC073 (D), CRC082 (E) and CRC090 (F) PDX models related to Figure 3 as indicated above.


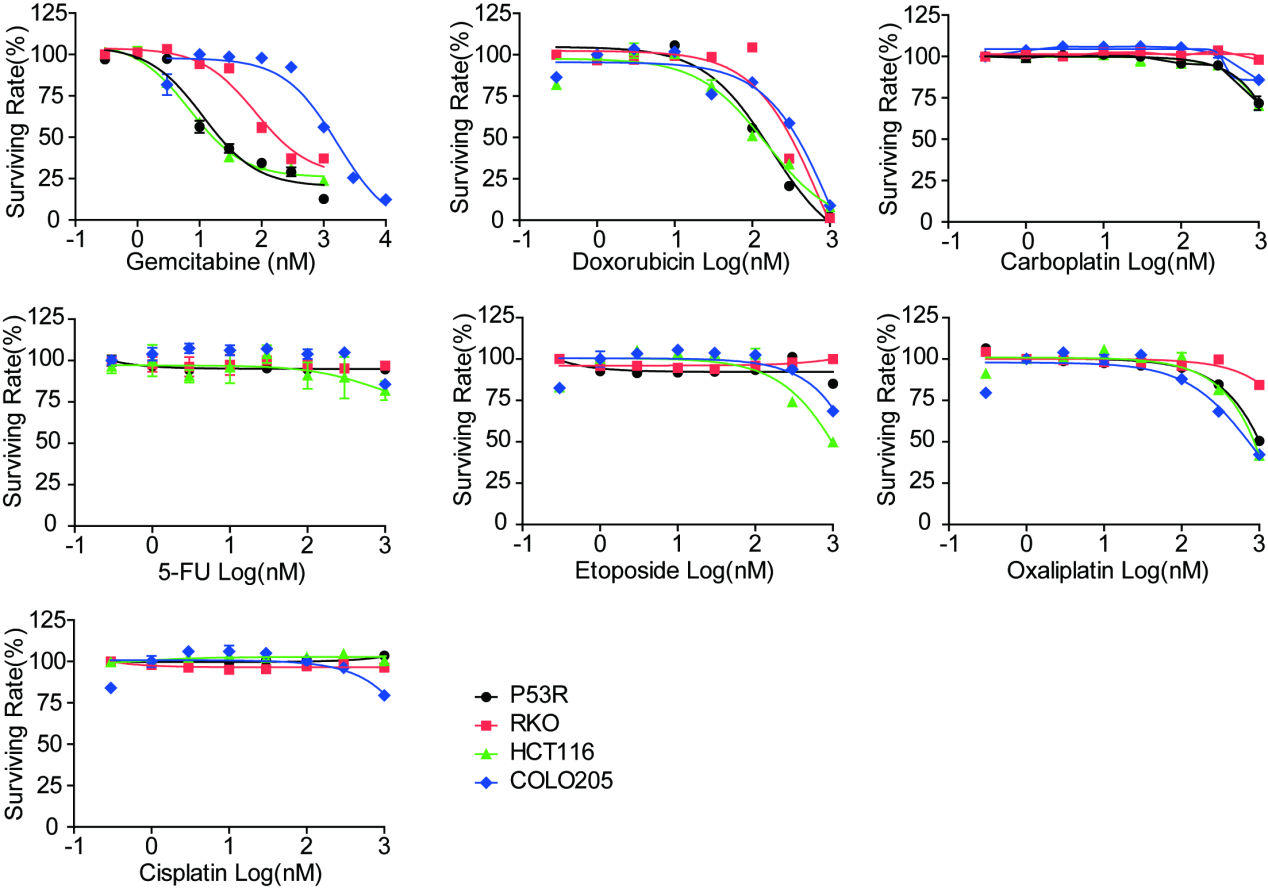


**Supplemental Figure 2. Cytotoxic effect of first-line chemotherapy drugs on CRC cells.** P53R, RKO, HCT116 and COLO205 cells were treated with gemcitabine, doxorubicin, carboplatin, 5-fluorouracil (5-FU), etoposide, oxaliplatin and cisplatin for 72 h, respectively. Cell survival was determined by CellTiter-Glo® Luminescent Cell Viability Assay. Each data point represents triplicate wells, with error bars representing ± SEM.


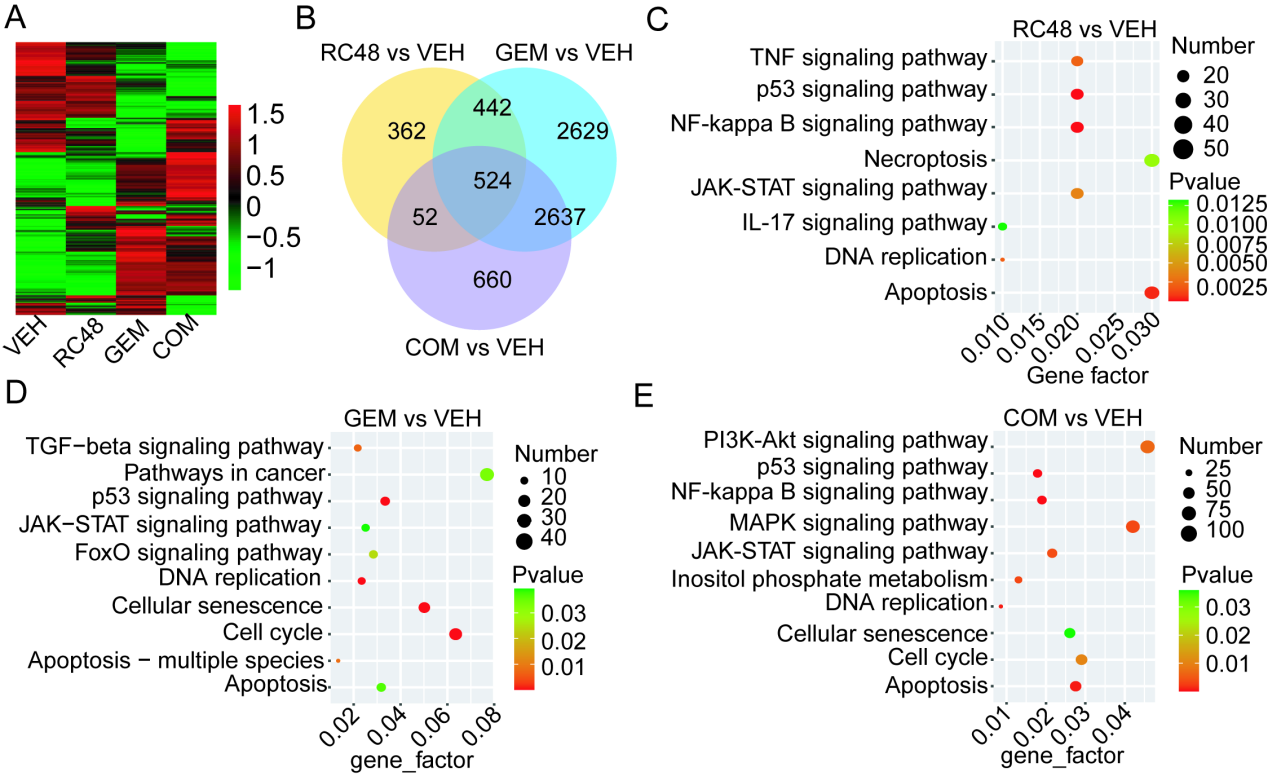


**Supplemental Figure 3. Synergic effects of RC48 and gemcitabine combinational treatment on the RKO cells**. (A) All differentially expressed genes (DEGs) in the vehicle-treated control (VEH; N=3), RC48 ( N=3), gemcitabine (GEM; N=3) and RC48 plus gemcitabine (COM; N=3) treated groups. (B) Numbers of overlapped DEGs in the RC48, gemcitabine (GEM), and RC48 plus gemcitabine (COM) treated samples as compared with the vehicle-treated samples (VEH). (C-E) Kyoto Encyclopedia of Genes and Genomes (KEGG) analysis of DEGs in the RC48 (C), GEM (D) and COM (E) treated samples as compared with the vehicle-treated samples in RKO cells (VEH).


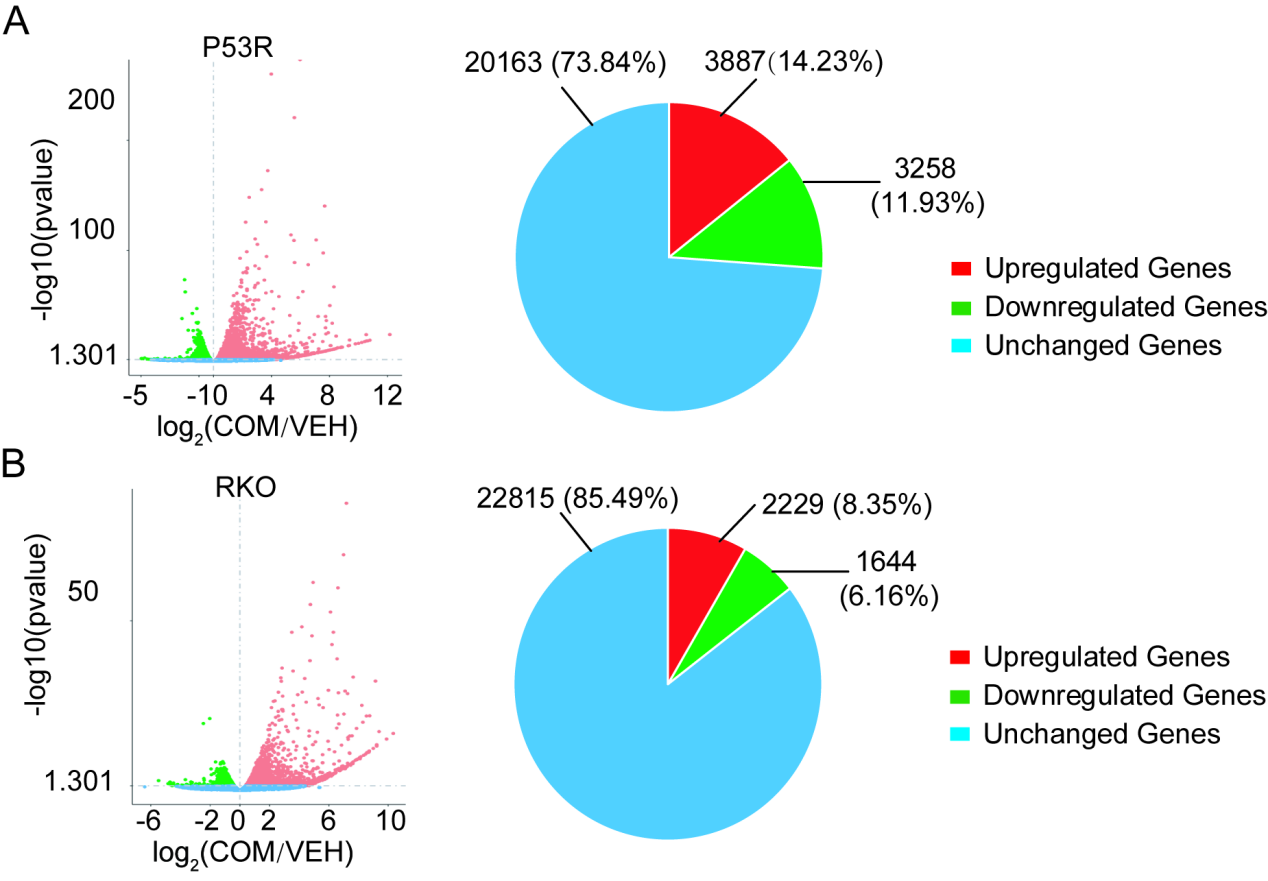


**Supplemental Figure 4. Combination of RC48 and Gemcitabine regulates the expression of genes by RNA-seq.** Volcano plot of the results of an RNA-Seq analysis showing the expression of DEGs in P53R (A) and RKO (B) cells between the control vehicle (VEH) and combinational treatment (COM). Upregulated, downregulated and unchanged genes are shown in red, green and blue, respectively. Values are presented as the log10 of tag counts.


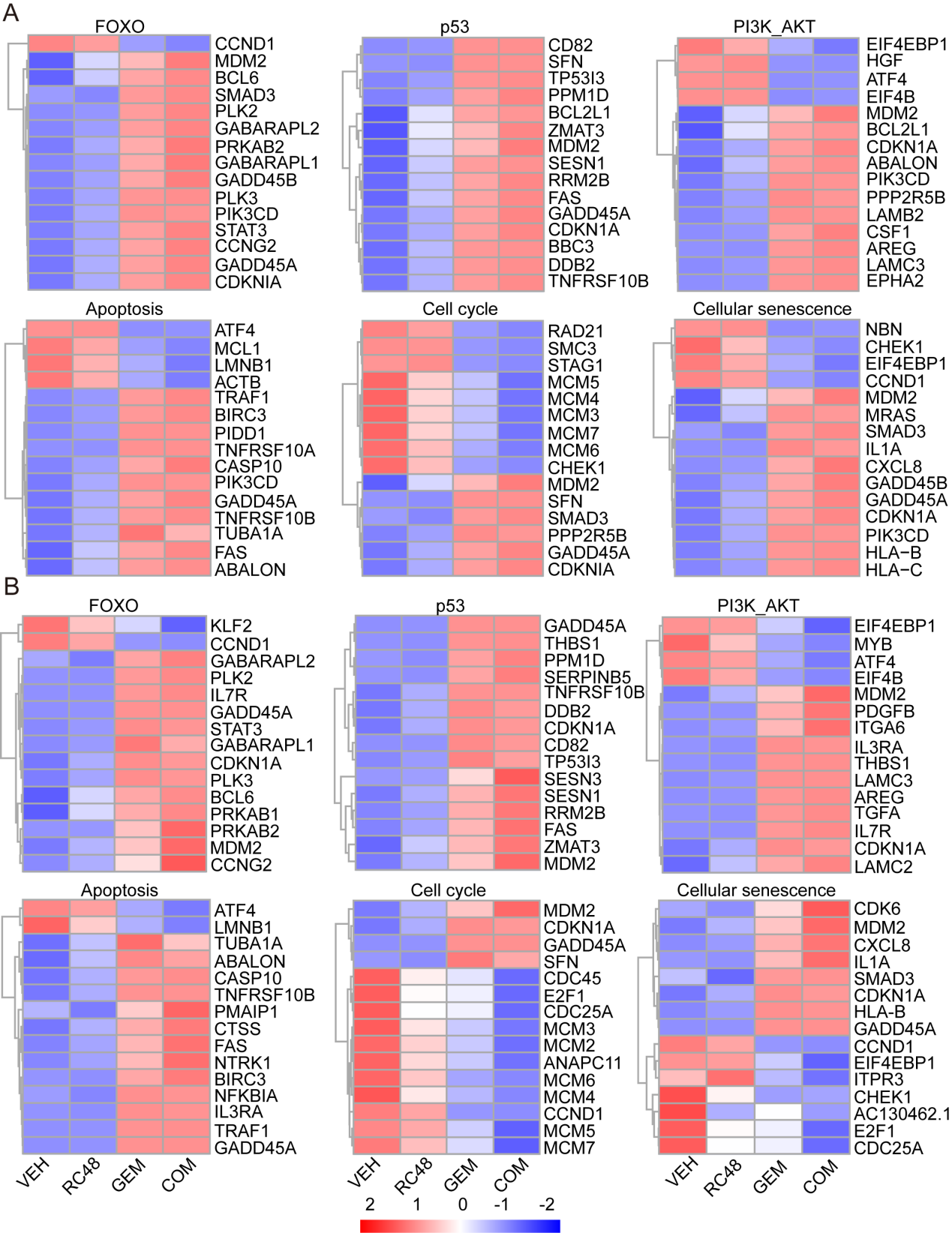


**Supplemental Figure 5. Combined therapy of RC48 and gemcitabine (GEM) significantly regulated DEGs expression of many pathways in CRC cells.** Heatmap of significantly regulated genes of transcriptomes in PR53R (A) and RKO (B) cells treated with the combination of RC48 and gemcitabine (COM), correlated with FOXO, p53, PI3K_AKT, apoptosis, cell cycle and cellular senescence pathways.


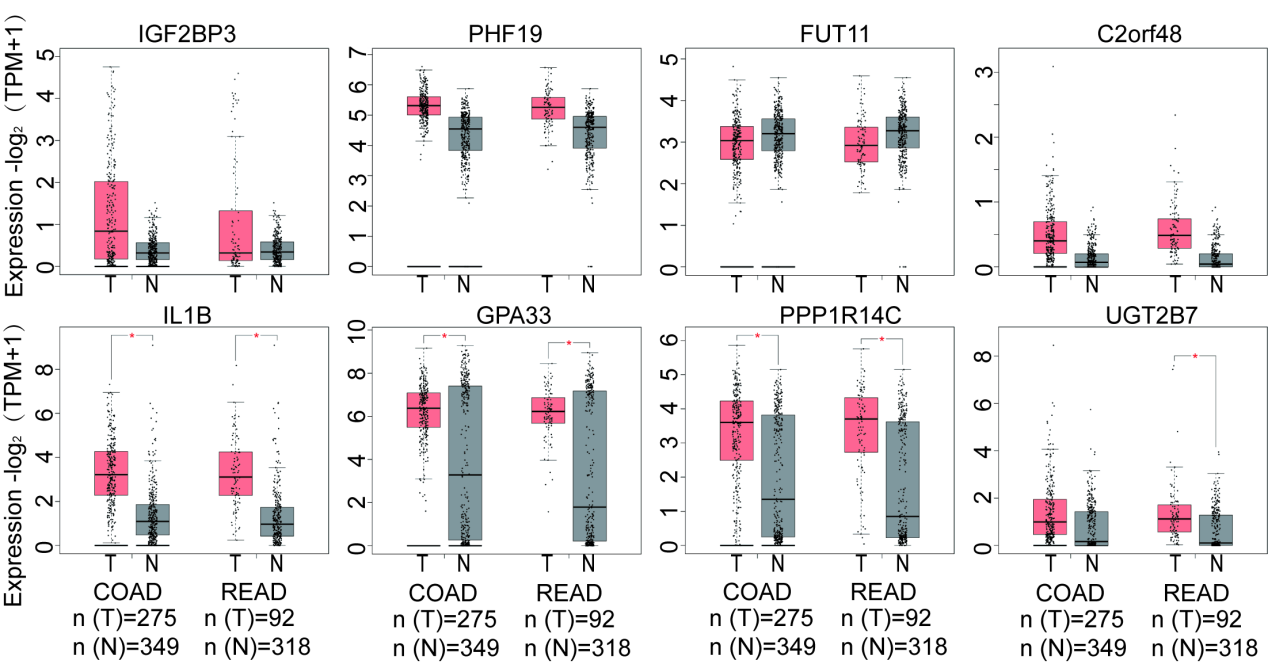


**Supplemental Figure 6.** The DEGs expression of IGF2BP3, PHF19, FUT11, C2orf48, IL1B, GPA33, PPP1R14C and UGT2B7 were shown in CRC in comparison to normal controls. p<0.05 is considered significant and was calculated by the two tailed Student’s t test.

**
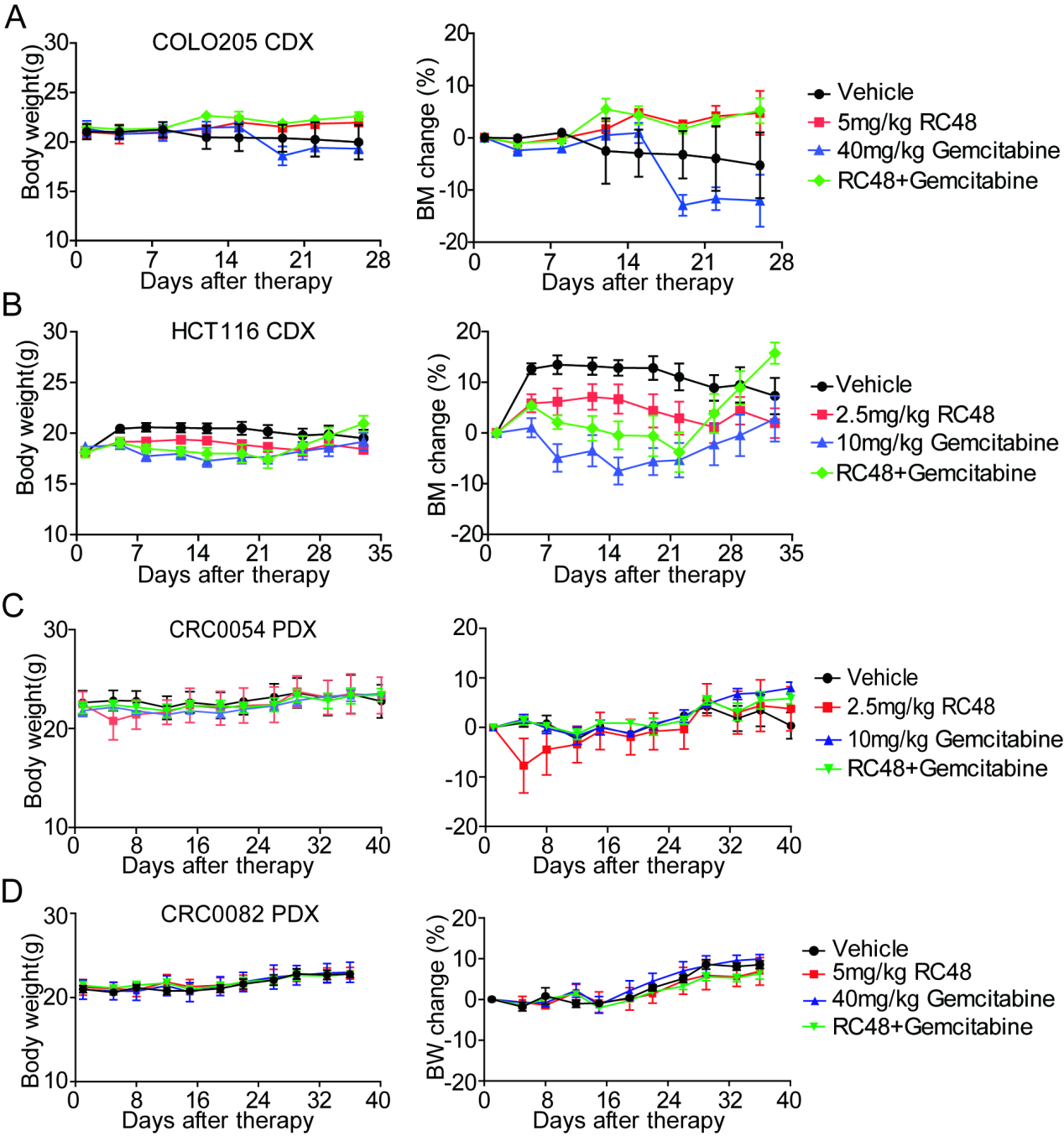
Supplemental Figure 7.** Mice body weights and the changes of the body weights were assessed twice every week in COLO205 (A), HCT116 (B) CDX and CRC054 (C), CRC082 (D) PDX models, related to Figure 7 as indicated above.


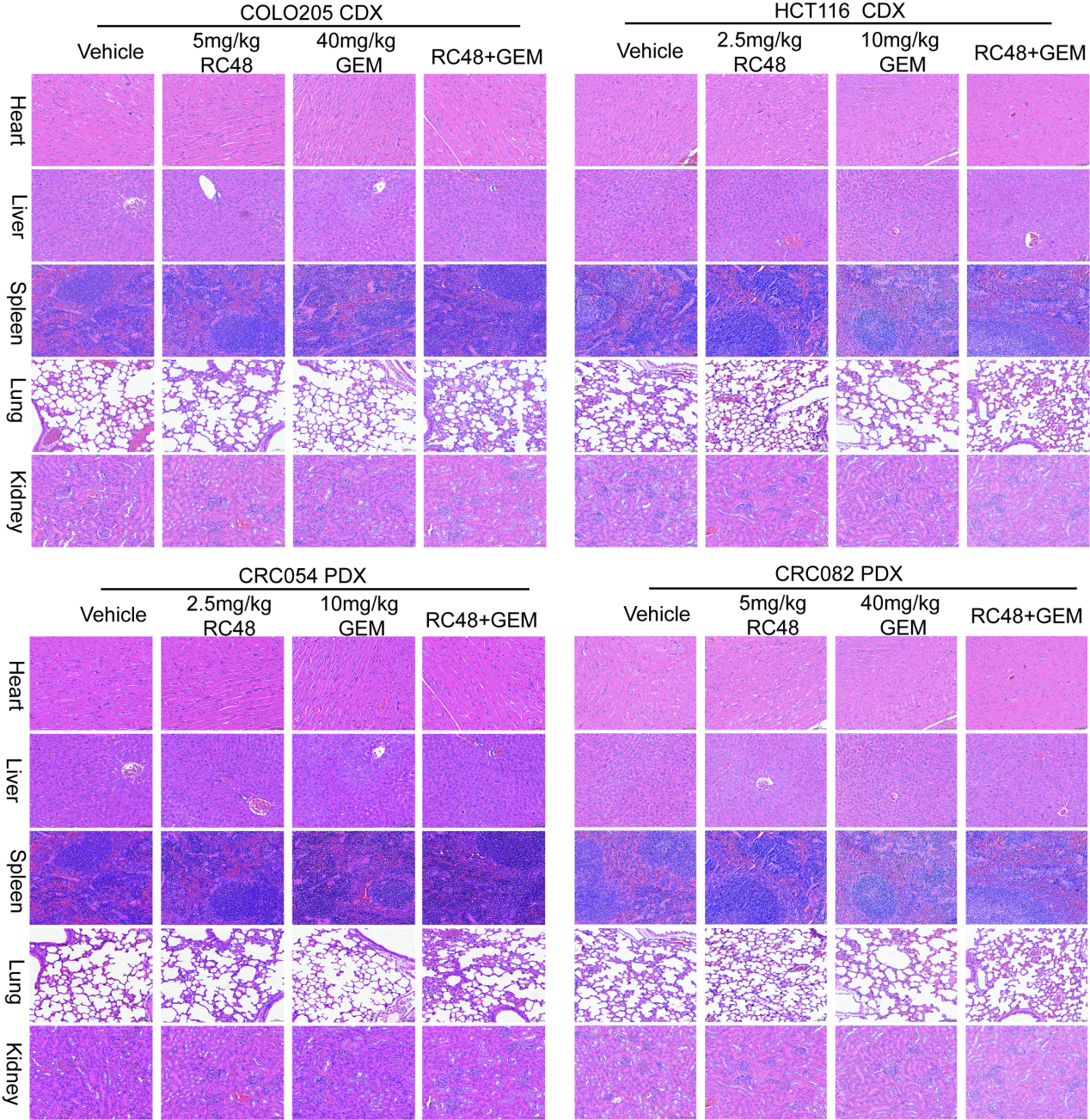


**Supplemental Figure 8**. Combination treatment of RC48 and gemcitabine (GEM) had no effects on heart, liver, spleen, lung and kidney in CDX and PDX models. H&E staining assay for the evaluation of pathological changes in these organs of these models, related to Figure 7. Images captured at 200× magnification. Scale bars = 50 µm.
